# Corticosteroids in Acute Lung Injury: The Dilemma Continues

**DOI:** 10.3390/ijms20194765

**Published:** 2019-09-25

**Authors:** Daniela Mokra, Pavol Mikolka, Petra Kosutova, Juraj Mokry

**Affiliations:** 1 Department of Physiology, Jessenius Faculty of Medicine in Martin, Comenius University in Bratislava, SK-03601 Martin, Slovakia; p.mikolka@gmail.com (P.M.); petra.kosutova@gmail.com (P.K.); 2 Biomedical Center Martin, Jessenius Faculty of Medicine in Martin, Comenius University in Bratislava, SK-03601 Martin, Slovakia; juraj.mokry@uniba.sk; 3 Department of Pharmacology, Jessenius Faculty of Medicine in Martin, Comenius University in Bratislava, SK-03601 Martin, Slovakia

**Keywords:** acute lung injury, acute respiratory distress syndrome, sepsis, corticosteroids, inflammation, oxidative stress, lung oedema

## Abstract

Acute lung injury (ALI) represents a serious heterogenous pulmonary disorder with high mortality. Despite improved understanding of the pathophysiology, the efficacy of standard therapies such as lung-protective mechanical ventilation, prone positioning and administration of neuromuscular blocking agents is limited. Recent studies have shown some benefits of corticosteroids (CS). Prolonged use of CS can shorten duration of mechanical ventilation, duration of hospitalization or improve oxygenation, probably because of a wide spectrum of potentially desired actions including anti-inflammatory, antioxidant, pulmonary vasodilator and anti-oedematous effects. However, the results from experimental vs. clinical studies as well as among the clinical trials are often controversial, probably due to differences in the designs of the trials. Thus, before the use of CS in ARDS can be definitively confirmed or refused, the additional studies should be carried on to determine the most appropriate dosing, timing and choice of CS and to analyse the potential risks of CS administration in various groups of patients with ARDS.

## 1. Introduction

Acute lung damage represents a life-threatening situation which can occur in all age groups. Diffuse alveolar injury, generation of lung oedema, neutrophil-mediated inflammation and ventilation-perfusion mismatch finally decrease lung compliance and cause profound hypoxemia [1]. Despite intensive research in the pre-clinical and clinical conditions, mortality of this disorder is alarmingly high. Some improvements were found for lung-protective ventilation, prone positioning, and administration of neuromuscular blocking agents (NMBA) [2]. Several other treatments, e.g., corticosteroids (CS) may also alleviate inflammatory changes and oedema of the injured lung. However, the results from experimental vs. clinical studies as well as among the clinical trials are often controversial. This article provides an overview of pathomechanisms of acute lung damage, similarities and differences of direct and indirect lung injury as well as pharmacological properties, mechanisms of action, and therapeutic potential of CS for acutely damaged lung. Both positive and potential adverse effects of CS administration in various animal models of ALI and in patients with ARDS are also discussed.

## 2. Acute Lung Injury

### 2.1. Definitions and Incidence

American-European Consensus Conference in 1994 [3] postulated following diagnostic criteria for acute lung damage in patients: (1) acute hypoxemia, defined as a ratio of arterial partial pressure of oxygen (PaO_2_) and fraction of inspired oxygen (FiO_2_), referred also as Horowitz index, with a value of PaO_2_/FiO_2_ < 200 mmHg (26.7 kPa) representing “acute respiratory distress syndrome” (ARDS) and PaO_2_/FiO_2_ between 200 mmHg (26.7 kPa) and 300 mmHg (40 kPa) representing “acute lung injury” (ALI); (2) finding of bilateral infiltrates on chest X-ray; (3) no increase in pulmonary artery wedge pressure.

Newer, so-called Berlin Definition from 2012 divided ARDS into three categories according to severity of hypoxemia to: mild (PaO_2_/FiO_2_ 200–300 mmHg), moderate (PaO_2_/FiO_2_ 100–200 mmHg) and severe (PaO_2_/FiO_2_ < 100 mmHg) forms of ARDS [4,5], while a value of PaO_2_/FiO_2_ was considered with a value of positive end-expiratory pressure (PEEP) of at least 5 cmH_2_O (0.5 kPa). The term “acute lung injury” used for the milder form of the lung damage in the older definition was omitted from the newer definition. Nowadays it is reserved for a general expression of the situation or for experimental studies where respiratory insufficiency is induced artificially and other clinically relevant signs except of hypoxemia cannot be determined.

Despite a substantial progress in understanding the pathophysiology and use of lung-protective ventilation techniques, the incidence of ARDS is still high, representing 30–80 cases per 100,000 population [6].

### 2.2. Aetiology of ARDS

ALI may develop from direct (pulmonary) reasons, e.g., in pneumonia, aspiration of the gastric content, near-drowning, inhalation of toxic gases, etc., or from indirect (extrapulmonary) reasons as a consequence of serious systemic injury, e.g., in sepsis, severe trauma with shock, or pancreatitis [7,8].

### 2.3. Pathophysiology of ARDS

The hallmarks of ARDS pathophysiology include dysregulated inflammation, inappropriate accumulation and activation of leukocytes and platelets, uncontrolled activation of coagulation pathways and altered permeability of alveolar-capillary barrier [9]. Progress of ARDS can be divided into three stages: exudative, proliferative and fibrotic. The initial or exudative phase (day 1–7) is characterized by a diffuse alveolar damage of epithelial and/or endothelial cells which release various factors contributing to the injury and cell death. The loss of integrity of alveolar-capillary barrier leads to flooding of the alveoli with proteinaceous fluid and dilution of pulmonary surfactant. Interstitial and intra-alveolar lung oedema decreases a lung compliance and impairs a gas exchange [10]. Damage to the lung tissue is associated with massive transmigration of immune cells into the diseased lung, as well. Activated neutrophils, alveolar macrophages, and fixed lung cells produce vast quantities of pro-inflammatory substances, e.g., interleukin (IL)-1β, IL-6, IL-8, tumour necrosis factor (TNF)α, and proteases, leading to further aggravation of the lung tissue injury [10,11]. Overproduction of oxidants and decrease in antioxidants lead to oxidation changes and cross-linking of proteins, lipids, DNA, and carbohydrates, deterioration of cell structures and their function, increased endothelial permeability and lung oedema formation, pulmonary epithelial dysfunction with impaired sodium ion transport and fluid reabsorption from the alveoli etc. [12]. In addition to intrinsically generated oxidants derived from phagocytic cells (recruited neutrophils and residential lung macrophages) and alveolar epithelial and endothelial cells [13], additional important source of oxidants is an inhalation of high oxygen concentrations used for mechanical ventilation of patients with severe ARDS [14]. 

Within several days the exudative phase fluently progresses to a proliferative phase that is characterized by resolution of pulmonary oedema and regeneration of damaged tissue by proliferation and phenotypic changes in type II alveolar cells, myofibroblasts and fibroblasts, and new matrix deposition. In the absence of recovery, the situation in some patients may progress to a fibrotic stage characterized by diffuse fibrosis and irreversible changes of lung architecture [10,15].

**Figure 1:**
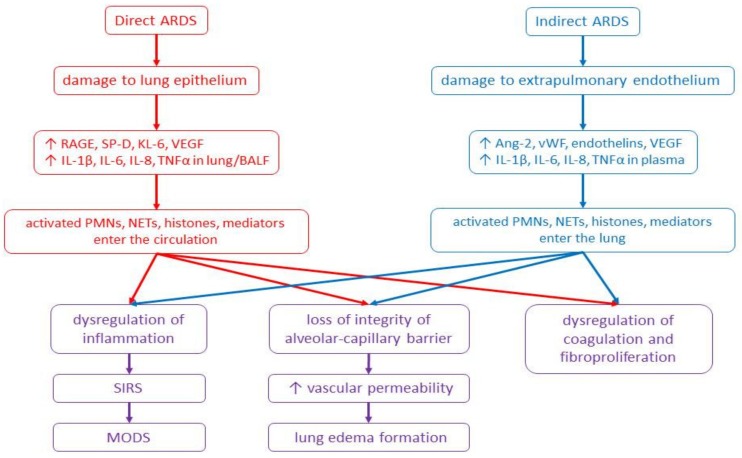
Scheme of pathomechanisms of direct and indirect forms of ARDS. Abbreviations: Ang-2: angiopoietin-2, ARDS: acute respiratory distress syndrome, BALF: bronchoalveolar lavage fluid, IL: interleukin, KL-6: Krebs von den Lungen-6, NETs: neutrophil extracellular traps, MODS: multiple organ dysfunction syndrome, PMNs: polymorphonuclears, RAGE: receptor for advanced glycation end-products, SIRS: systemic inflammatory response syndrome, SP-D: surfactant protein D, TNFα: tumour necrosis factor alpha, VEGF: vascular endothelial growth factor, vWF: von Willebrand factor, ↑: increase.

In direct lung injury, the noxious stimulus primarily hits the lung structures. Activation of the innate immune response by binding of microbial products or cell-injury associated endogenous molecules (danger-associated molecular patterns, DAMPs) to pattern recognition receptors (e.g., Toll-like receptors) on the lung epithelium and alveolar macrophages triggers an acute lung inflammation [9]. As additional immune effector mechanisms contributing to the tissue injury, neutrophil extracellular traps (NETs), extracellular histones, and granular proteins (e.g., neutrophil elastase and myeloperoxidase) formed by dying neutrophils have been identified. The released histones, major proteins of chromosomes, are highly cytotoxic and act as DAMPs, further inducing epithelial and endothelial cell death. When entering the circulation, histones stimulate a platelet aggregation, promote a recruitment of neutrophils, and aggravate a systemic inflammation [16,17]. The systemic leukocyte activation can progress to systemic inflammatory response syndrome (SIRS), multiple organ dysfunction syndrome (MODS), and multiple organ failure [18]. If the primary cause of ARDS is located in other tissues, lung inflammation and oedema formation may be triggered by high concentrations of histones to which is the lung highly susceptible [16,19]. However, many other substances, like pro-inflammatory cytokines TNFα and IL-1β, high-mobility group box 1 protein, or mitochondrial DNA, also act as DAMPs and induce lung inflammation and ARDS [20] (Figure 1).

There are additional differences between the direct and indirect forms of ALI/ARDS, as well. In the direct ALI/ARDS, the injury is more localized to the alveolar epithelial cells, with alveolar collapse, accumulation of neutrophils, fibrin deposition, formation of hyaline membranes and alveolar wall oedema. In the indirect ALI/ARDS, the injury to endothelial cells is more diffuse, and typical is the finding of interstitial oedema and smaller lung accumulation of neutrophils than in the direct form. Moreover, in the direct form of ALI/ARDS, concentrations of pro-inflammatory cytokines TNFα, IL-1β, IL-6 and IL-8 increase in the bronchoalveolar lavage fluid (BALF) or lung tissue homogenates. In the indirect form of ALI/ARDS, increased cytokines levels are detected predominantly in the plasma indicating that the lung injury originates due to the action of mediators released from extrapulmonary foci into the systemic circulation [21,22]. Regarding a primary injury to the epithelial cells in the direct ALI/ARDS, surfactant protein D has been identified as a valuable marker of injury to type II alveolar cells and receptor for advanced glycation end products (RAGE) as a marker of type I alveolar cells injury. Damage of endothelial cells and systemic inflammation which are more prominent in the indirect ALI/ARDS can be confirmed by increased plasma levels of von Willebrand factor (vWf), IL-6, IL-8 and angiopoietin-2 [10,23].

### 2.4. Therapeutic Options in ARDS

Despite the understanding of ARDS pathophysiology has greatly improved during last decades, the efficacy of used therapeutic approaches is limited.

The currently recommended therapy is based on so-called lung-protective mechanical ventilation ensuring adequate oxygenation and elimination of CO_2_ in minimized additional damage from mechanical ventilation. Ventilation of the lungs with excessive volumes or pressures in inappropriately low PEEP leads to alveolar damage and inflammatory response called “ventilator-induced lung injury (VILI) [2,24]. However, VILI can also aggravate the existing lung injury originated from other reasons. The ventilation protecting the lung from VILI should use low tidal volumes (V_T_ < 6 mL/kg of the predicted body weight) combined with limited inspiratory plateau pressures (<30 cm H_2_O) to prevent a lung overdistension (“barotrauma/volutrauma”). The ARMA trial published in 2000 showed that lower tidal volume ventilation (V_T_ of 6 mL/kg, plateau airway pressure of 30 cm H_2_O) compared to “traditional” strategy with V_T_ of 12 mL/kg and plateau pressure of 50 cm H_2_O resulted in lower mortality, more ventilator-free days and more days free of non-pulmonary organ failure [25]. Reduced mortality and improved outcomes in early use of lung protective ventilation have been confirmed also by the recent studies [26,27]. In addition, the lung-protective ventilation should use levels of PEEP appropriately titrated on individual patients according to PEEP/FiO_2_ table using the level of saturation/oxygenation as a target to prevent repetitive opening and closing of the terminal lung units (“atelectrauma”). In too low PEEP, a part of recruitable tissue may collapse while excessive PEEP may increase dead volume and tissue stretch [2,28,29]. For re-inflation of the collapsed lung regions, different types of recruitment manoeuvres, such as sustained inflation, intermittent sighs, and stepwise increase in inspiratory pressure could be used, however, the optimal recruitment method has not been defined yet [2,30].

Further improvement may be observed for prone positioning, which recruits a lung parenchyma, especially in the acute phase of severe ARDS [29,31]. This approach enables better ventilation/perfusion matching with a consequent improvement in CO_2_ clearance, more homogenous distribution of ventilation with reduction of VILI and recruitment of dorsal regions through the redistribution of lung densities [32,33].

For pharmacotherapy of ALI/ARDS, several groups of medicaments have been successfully used [34,35], although a positive response to some of them has been shown just in specific subgroups of patients [1,6]. Among the pharmacological interventions, use of NMBA seems to be the most promising. As spontaneous breathing in the patients with severe ARDS might generate high transpulmonary pressure, use of NMBA enables amelioration of patient-ventilator synchrony and reduces oxygen consumption leading to improved survival [36,37].

Improvements in duration of mechanical ventilation, duration of hospitalization or oxygenation have been observed also for CS, exerting a wide spectrum of potentially desired effects including anti-inflammatory, antioxidant, pulmonary vasodilator, and anti-oedematous actions. In the following subsections, mechanisms of CS action and response to their delivery in various forms of direct and indirect ALI in animal models and in patients with ARDS are discussed.

## 3. Corticosteroids (CS)

### 3.1. Mechanisms of CS Action

Effects of CS are mediated by both genomic and non-genomic mechanisms. The classical genomic mechanism is mediated via cytosolic glucocorticoid receptor (cGCR) and involves many steps; therefore, these effects of CS cannot be observed earlier than 4 h after delivery. Thanks to their lipophilic structure, CS easily pass through plasma membranes and bind to ligand-binding domain of cGCR. The glucocorticoid-cGCR complex translocates to the nucleus within 10–30 min of cell exposure to CS and binds to DNA-binding sites termed glucocorticoid response elements (GRE). Binding to the positive GRE, the glucocorticoid-cGCR complex activates a transcription of anti-inflammatory proteins (such as IL-10, annexin 1, inhibitor of NF-κB, etc.) and regulator proteins important for metabolism, whereas this process (“transactivation”) is likely responsible also for numerous adverse effects [38,39]. Binding to the negative GRE, the glucocorticoid-cGCR complex inhibits transcription of inflammatory transcription factor proteins such as nuclear factor-κB (NF-κB) and activator protein-1 (AP-1). This process called “transrepression” is responsible for major anti-inflammatory and immunosuppressive effects characterized by suppressed synthesis of pro-inflammatory cytokines (IL-1, TNFα, interferon (IFN)γ, etc.) [40], inhibited expression of nitric oxide (NO) synthase (NOS) and production of NO [41], down-regulated transforming growth factor (TGF)β, important for cellular differentiation of fibroblasts to myofibroblasts, extracellular matrix deposition, and impairment of epithelial repair [42].

The non-genomic mechanisms of CS action involve putative membrane-associated receptors and second messengers. These effects have rapid onset (seconds to minutes) and short duration of action (60–90 min) [43]. Non-genomic interactions can be classified into three categories: (1) non-specific interactions of CS with cellular membranes which change physicochemical properties of the membrane, mineral transport across the cell membrane, and production of adenosine triphosphate (ATP) leading to immune cell suppression [39,44]; (2) specific interaction with membrane-bound glucocorticoid receptors (GRs) that is responsible for rapid T-cell immunosuppressive action [45]; and (3) non-genomic binding to cytosolic GRs and release of heat shock protein (HSP) 90 decreasing arachidonic acid production [46]. Non-genomic mechanism can be also involved in activation of endothelial NOS (eNOS). Binding of CS to GRs stimulates phosphatidylinositol-3′-kinase and Akt kinase, leading to eNOS activation and NO-dependent vasorelaxation [39].

In both ARDS and sepsis, systemic inflammation is activated by NF-κB signalling system and down-regulated by activated glucocorticoid receptor-α (GRα). However, in ARDS both expression and nuclear translocation of GRα decrease [47]. Due to insufficient GRα-mediated (and endogenous glucocorticoid activated) downregulation of proinflammatory actions of NF-κB, the concentrations of various markers of inflammation, haemostasis, and tissue repair increase in both plasma and BALF, despite elevated levels of circulating cortisol [48]. Lung injury can be even aggravated when synthesis of endogenous glucocorticoids is inhibited [49]. On the other hand, restoring GRα number and function after CS treatment can accelerate the resolution of lung inflammation and oedema, restoration of alveolar-capillary membrane integrity and lung tissue homeostasis, and protect the lung from an additional injury [50,51].

### 3.2. Corticosteroids in Experimental Models of ALI

The response to administered CS has varied among different types of lung damage and used animal models of ALI. Models of direct ALI can be evoked by inhalation of noxious chemicals, i.t. instillation of LPS, repetitive saline lung lavage, excessive mechanical ventilation etc. Various degree and special characteristics of the individual types of lung injury can be therefore responsible for different responses to CS treatment.

As reviewed by De Lange and Meulenbelt [52], use of CS in direct ALI caused by inhalation of poorly water-soluble chemical agents (nitrogen dioxide, ozone, phosgene etc.) or water-soluble chemical agents (chlorine, ammonia etc.) showed no clear benefit in the acute phase of lung injury. In the recovery phase, the effect of CS was even harmful, whereas CS hindered proliferation of type II alveolar cells and their differentiation into type I cells, important for alveolar re-epithelialization and water removal from the alveoli [52]. In phosgene-injured animals, CS treatment showed no change in mortality, lung oedema, or shunt fraction [53,54], except of some improvements in cardiac stroke volume [53], or even aggravated lung oedema [55]. Similarly, budesonide inhalation did not improve respiratory functions in rabbits with aerosolized ammonia-induced ALI [56]. On the other hand, early treatment with i.v. methylprednisolone enhanced respiratory mechanics and prevented changes in tissue impedance and extracellular matrix [57], increased oxygenation, reduced number of inflammatory cells in BALF, and decreased lung injury score (LIS) in rats with paraquat-induced ALI [58]. Treatment with CS was of benefit also in ALI caused by chlorine inhalation. Mometasone and budesonide given intraperitoneally (i.p.) 1 h after chlorine inhalation dose-dependently inhibited neutrophil influx into the lung tissue and BALF and reduced pulmonary oedema [59]. Intramuscular (i.m.) budesonide prevented influx of M2 macrophages and development of airway fibrosis and hyperreactivity in mice [60]. In pigs, nebulized beclomethasone dipropionate improved oxygenation, partially prevented an increase in pulmonary vascular resistance and a decrease in lung compliance [61], while inhaled budesonide enhanced the lung functions [62], and both inhaled budesonide and i.v. betamethasone improved blood gases decreased the wet-dry lung weight (WD) ratio and had a tendency to improve the lung histology [63].

Positive results for CS have been also observed in models of surfactant depletion representing the other type of direct ALI. In ALI induced by i.t. instillation of seawater in rabbits, dexamethasone (1 mg/kg i.v.) improved oxygenation and thoracic compliance, reduced LIS and inflammatory cells infiltration, and decreased lung concentrations of myeloperoxidase (MPO) and TNFα [64]. Similarly, in rabbits with ALI induced by repetitive saline lung lavage, i.v. dexamethasone [65] and i.t. budesonide [66] enhanced gas exchange, and alleviated inflammation and histopathological signs of the lung injury.

In addition to inhalation of noxious agents, the lung can be injured by excessive mechanical ventilation using high tidal volumes and/or high insufflation pressures [2]. In rats ventilated with high tidal volumes, dexamethasone (6 mg/kg i.p.) attenuated pulmonary and cardiovascular injury, prevented increase in serum levels of aspartate aminotransferase, nitrates and nitrites, IL-6, and expressions of cyclooxygenase-1 and cyclooxygenase-2 in the heart [67]. Similarly, lung injury, inflammation, apoptosis, and lung oedema caused by large volume ventilation in rats were ameliorated by i.t. budesonide as indicated by enhanced PaO_2_/FiO_2_, decreased WD ratio, total protein, neutrophil elastase level, and neutrophil count in BALF, decreased TNFα, IL-1β, IL-6, intercellular adhesion molecule (ICAM)-1, and macrophage inflammatory protein (MIP)-2 and increased IL-10 levels in BALF and plasma, inhibition of phosphorylated NF-kB levels in the lung tissue, mitigated lung histological changes and apoptosis shown by down-regulated Bax, caspase-3, and cleaved caspase-3 and up-regulated Bcl-2 [68].

In various animal models of i.t. LPS-induced ALI, pretreatment with dexamethasone [69] or budesonide [70] prevented formation of lung oedema and alleviated lung inflammation indicated by reduced translocation of NF-κB and lower concentrations of cytokines and chemokines. Similarly, treatment with i.p. dexamethasone at a dose of 10 mg/kg reduced number of neutrophils in BALF, and decreased pro-inflammatory cytokines in the lung and oxidative markers in BALF in mice exposed to aerosolized LPS [71]. In i.t. LPS-injured mice, methylprednisolone (2 mg/kg i.v. at 6 h, 24 h or daily for seven days) enhanced lung mechanics, reduced fibroelastogenesis, and prevented an increase in matrix metalloproteinases (MMP), suggesting a potential of CS for prevention of fibrotic changes [72]. In rats with LPS-induced ALI, pulmonary fibrosis occurred in parallel with inflammation, whereas dexamethasone alleviated the inflammation and fibrosis parameters and elevated GR expression in the lung, probably via upregulating GR levels and promoting the nuclear translocation of GR protein [73]. Favourable results for i.t. budesonide were also demonstrated in rats with combined indirect and direct ALI model induced by i.v. endotoxin and ventilator-induced lung injury. Budesonide treatment enhanced PaO_2_/FiO_2_, decreased total cell count, macrophages, and neutrophils in BALF, and levels of ICAM-1, TNFα, IL-1β, and IL-8 in BALF and serum, elevated levels of IL-10 in BALF and serum, reduced histopathological signs of lung injury and apoptosis, and improved survival [74].

CS have been also tested in models resembling indirect form of ARDS or sepsis. In pigs with endotoxemia, pretreatment with i.v. methylprednisolone prevented an impairment in gas exchange and production of lung oedema and improved cardiac output. Furthermore, the improvement in gas exchange and lung oedema formation were observed also for methylprednisolone given 2 h after endotoxin infusion [75]. Contrary, i.v. delivery of methylprednisolone which started 30 min before i.v. infusion of oleic acid and continued for 6 h did not prevent degradation of surfactant in BALF nor suppressed phospholipase A_2_ activity, but reduced IL-8 in plasma and BALF and slightly improved LIS [76]. On the other hand, treatment with both i.t. and i.v. CS prolonged survival in experimental septic shock [77]. In septic pigs, nebulized beclomethasone improved oxygenation, decreased venous admixture, and increased lung compliance compared to non-treated controls [78]. In a rabbit model of i.v. endotoxin-induced lung injury, i.t. budesonide enhanced lung compliance and PaO_2_/FiO_2_, decreased WD ratios, total protein, neutrophil elastase, white blood cells and the percentage of neutrophils in BALF, decreased TNFα, IL-1β, and IL-8 and increased IL-10 in BALF, reduced lung injury and improved survival rate [79]. In guinea pigs with LPS-induced sepsis, where mRNA and protein levels of GRα decreased and protein expression of GRβ increased in the injured lungs, high-dose methylprednisolone (40 mg/kg i.p.) administered simultaneously with LPS potentiated the decrease in expression of GRα and influenced expression of GRβ what impaired GRα nuclear translocation, but strongly blocked sepsis-induced NF-κB activation and transmigration of inflammatory cells into BALF [47]. These findings confirmed the hypothesis that prolonged CS-induced downregulation of the inflammatory response can be associated with an improvement in the pulmonary and likely also in the extrapulmonary functions [51]. 

In a canine model of septic shock, dexamethasone given immediately after bacterial challenge reversed shock, improved survival, and reduced the pulmonary and cardiovascular dysfunction [80]. Similarly, in rats with i.v. LPS-induced endotoxic shock, dexamethasone down-regulated both NF-κB DNA-binding activity and expression of p65 protein in the nuclear brain extracts suggesting that NF-κB activation and nuclear translocation of NF-κB play a significant role in the brain tissue injury in endotoxic shock, which can be alleviated by dexamethasone [81].

### 3.3. Corticosteroids in Patients with ARDS

The administration of CS for prevention and treatment of ARDS has been intensively discussed for decades. Data from clinical trials are often conflicting because of differences in patients selection, methods of CS delivery (timing of initiation, used pharmacological agents, dosing, duration of treatment) and data processing. Therefore, no clear conclusions on the use of CS in ARDS have been established until now.

In 1980s, randomized controlled trial (RCT) on short course of high dose methylprednisolone (120 mg/day) delivered in the early ARDS showed no effect on mortality, occurrence of infectious complications or ventilatory parameters [82] (Table 1). This lack of effect could be attributed to suppression of HPA axis and higher risk of infection.

However, more recent studies have indicated that a prolonged CS delivery can be of benefit. In the pilot study by Meduri et al. from 1995 [93], prolonged treatment with methylprednisolone (initial bolus of 200 mg i.v., then every 6 h at a dosage of 2–3 mg/kg/day until extubation, after extubation oral methylprednisolone or prednisone slowly tapered, total duration of treatment 6 weeks) in 9 patients with late ARDS decreased TNFα and IL-6 in the plasma of both rapid and delayed responders by day 7, while IL-1β decreased by day 5 in rapid responders and by day 10 in delayed responders. Decline in plasma and BAL cytokines was associated with improvement in LIS and BALF albumin [93].

In the following RCT by Meduri et al. which included patients with unresolving ARDS, treatment with methylprednisolone (initial dose 2 mg/kg/day, duration of treatment 32 days) in 16 patients improved lung injury and MODS scores, enhanced extubation and reduced mortality, with similar rate of infections per day of treatment to placebo-treated group [83] (Table 1). Further analyses of blood samples showed that methylprednisolone-treated patients exerted obvious reductions of TNFα, IL-1β, IL-6, adrenocorticotropic hormone (ACTH) and cortisol concentrations over time. Normal peripheral blood leukocytes exposed to plasma samples of methylprednisolone-treated patients showed enhanced GR-mediated activity and suppression in NF-κB DNA-binding and transcription of TNFα and IL-1β, supporting the hypothesis of inadequacy of endogenous glucocorticoid-mediated control of inflammation and systemic inflammation-induced peripheral glucocorticoid resistance in ARDS [94].

Not so encouraging results were found by Steinberg et al. [85] in a multi-centre RCT on CS use in patients with persistent ARDS. Methylprednisolone-treated group had similar mortality rate at 60 and 180 days, but increased 60- and 180-day mortality rates at least 14 days after the onset of ARDS. However, methylprednisolone elevated the number of ventilator-free and shock-free days during the first 28 days what was associated with improved oxygenation, lung compliance, and blood pressure with fewer days of vasopressor therapy. Methylprednisolone did not increase the rate of infections but increased the rate of neuromuscular weakness. The lack of benefits of this therapeutic protocol could be related to too late CS administration or rapid CS weaning. Considering the results of the RCTs by Meduri et al. [83] and Steinberg et al. [85], the routine use of methylprednisolone for persistent ARDS, particularly when starting methylprednisolone therapy more than two weeks after the onset of ARDS, was not further supported.

Administration of CS in the early phase of ARDS has appeared as more promising. RCT by Meduri et al. from 2007 on administration of low-dose methylprednisolone in early severe ARDS [87] showed a significant decrease in duration of mechanical ventilation and ICU stay, reduced mortality and lower occurrence of infectious complications (Table 1).

These findings were supported also by meta-analyses of clinical trials performed before 2010 which postulated that despite methodological limitations some “weak” recommendation of low-to-moderate dose of CS for ARDS < 14 days duration can be made because of a trend toward reduction of mortality, increased number of ventilator-free days, reduced length of intensive care unit (ICU) stay, improved MODS and LIS scores and PaO_2_/FiO_2_ ratio in use of low-dose CS, without higher risk of adverse reactions. On the other hand, preventive CS administration was associated with a trend to increase both the odds of patients developing ARDS and the risk of mortality in those who subsequently developed ARDS [95,96,97].

In the following decade, positive effects of CS treatment in early ARDS have been observed in more recent studies. Seam et al. in their retrospective analysis of data demonstrated that methylprednisolone treatment improved LIS, shortened duration of mechanical ventilation, and reduced ICU mortality in early ARDS compared to non-treated subjects [98]. Methylprednisolone decreased plasma concentrations of IL-6, an important biomarker of inflammation and prognosis, by days 3 and 7 in patients with direct ARDS, but only at day 3 in patients with indirect ARDS. Levels of protein C, an endogenous anticoagulant linking coagulation and inflammation cascades, elevated with methylprednisolone on days 3 and 7 in patients with infectious and/or pulmonary form of ARDS but not in patients with non-infectious or extrapulmonary origins of ARDS. Levels of proadrenomedullin (a prohormone of adrenomedullin which elevated levels are associated with poor outcome) decreased with methylprednisolone on day 3 in patients with infectious or extrapulmonary ARDS but not in non-infectious or pulmonary ARDS [98].

As reviewed in 2016 by Schwingshackl and Meduri [51], data from eight controlled trials (*n* = 622) demonstrated an increase in mechanical ventilation-free days and ICU-free days by day 28 of CS therapy in ARDS, and increased hospital survival if treatment initiated before day 14 of ARDS. Prolonged CS treatment was not associated with increased risk for nosocomial infections [51]. More recent meta-analysis including nine RCTs investigated low-to-moderate dose prolonged CS treatment in ARDS. This therapy was considered as safe, reducing the time to endotracheal extubation, and mortality, and increasing number of ventilation-free days, shortening ICU and hospitalization stay. Therefore, administration of methylprednisolone in early moderate-to-severe (1 mg/kg/day) and late persistent ARDS (2 mg/kg/day) was suggested for promising in patients with early moderate-to-severe ARDS [99]. Nevertheless, opposite results were published for high-dose CS given within seven days of admission. The retrospective and observational Japanese study showed increased mortality rates within 3 months compared to the non-high-dose CS group, indicating that high-dose CS treatment is not suitable for patients with ARDS [100].

Among the human studies, there are only few containing a systematic information on CS treatment in ARDS caused by inhalation of chemical agents and the data are often inconsistent, because the human data are almost limited to accidental exposures [52].

Low-dose CS could be of benefit also in patients with severe pneumonia, however, the results of trials are conflicting [101]. In the RCT included patients admitted to ICU with severe community-acquired pneumonia (CAP), treatment with hydrocortisone (200 mg i.v. bolus followed by an infusion of 10 mg/h for seven days) improved PaO_2_/FiO_2_ and MODS score by day 8, reduced concentrations of C-reactive protein (CRP), delayed septic shock, reduced duration of hospital stay and mortality [84] (Table 1). In a retrospective, observational study of a cohort of patients hospitalised with severe CAP, systemic administration of CS was independently associated with reduced mortality [102]. In a double-blind, placebo-controlled trial, treatment with dexamethasone (5 mg i.v. once a day) in 151 adults with CAP for four days from admission shortened duration of hospital stay, but higher percentage of CS-treated patients had hyperglycaemia [88] (Table 1). Contrary, as demonstrated in RCT by Snijders et al., delivery of 40 mg prednisolone for seven days was associated with increased late failure and did not improve the outcome of patients with CAP, therefore, prednisolone should not be recommended as a routine adjunctive treatment in CAP [103]. Non-uniform data have been also found in the meta-analyses on CS use in CAP. The meta-analysis from 2013 showed that there is no positive effect on mortality or clinical stability in adult patients with CAP treated with systemic CS; even, CS administration was associated with prolonged length of stay [104]. Contrary, an updated analysis of 17 RCTs published in 2017 comprising of 2264 participants assessed systemic CS therapy for adults (*n* = 1954) and children (*n* = 310) with CAP, with or without healthcare-associated pneumonia (HCAP) showed that CS reduced mortality in adults with severe pneumonia, but not in adults with mild-to-moderate pneumonia. CS reduced early clinical failure rates in patients with severe and mild-to-moderate pneumonia, shortened duration of hospital and ICU stays, development of respiratory failure or shock not present at pneumonia onset and rates of pneumonia complications. Among children with bacterial pneumonia, CS decreased early clinical failure rates and reduced time to clinical cure. Except of more frequent hyperglycaemia in adults no significant differences between CS-treated patients and controls for other adverse events or secondary infections were observed [105].

The effects of CS on clinical outcomes of patients with influenza pneumonia are also conflicting. Recent meta-analysis of data from 6548 adult patients with influenza pneumonia showed significant heterogeneity in outcome measures and association of CS therapy with higher mortality, longer ICU stay, and a higher rate of secondary infection [106].

Rather encouraging results were observed in RCT on paediatric ARDS where children were administered methylprednisolone (loading dose of 2 mg/kg and continuous infusions of 1 mg/kg/day on days 1–7 and then tapered over days 8–14). Despite higher plateau pressures on days 1 and 2 due to worsened lung compliance, CS-treated patients had lower PaCO_2_, higher pH and higher PaO_2_/FiO_2_ ratios compared with the placebo group. Lower number of CS-treated patients required treatment for postextubation stridor or supplemental oxygen at ICU transfer, while CS therapy was not linked with obvious side effects [107] (Table 2).

Additional analyses of blood samples of children with ARDS treated with methylprednisolone showed a decrease in levels of IFN-α, IL-6, IL-10, monocyte chemoattractant protein (MCP)-1, granulocyte colony-stimulating factor (G-CSF), and granulocyte macrophage colony-stimulating factor (GM-CSF) and increased IL-17α by day 7 and increased total leukocyte and platelets counts by day 7 [108]. By day 7, methylprednisolone also reduced the plasma concentrations of MMP-8 (indicating reduced activation of neutrophils), prevented an increase in soluble ICAM-1 (indicating decreased endothelial injury), and decreased soluble RAGE (indicating epithelial injury and recovery) what correlated well with the respiratory functions of children with ARDS [109].

CS have been also used in the treatment of sepsis and septic shock. Similarly to direct ARDS, results of the trials are often contradictory [110]. In a prospective, randomized study, 41 patients with early hyperdynamic septic shock were treated with low-dose hydrocortisone (50 mg bolus followed by a continuous infusion of 0.18 mg/kg b.w./h, dose reduced after shock reversal to 0.06 mg/kg/h and afterward slowly tapered). The hydrocortisone treatment shortened the time to cessation of vasopressor support and decreased plasma levels of IL-6 indicating haemodynamic and immunomodulatory effects of low-dose CS accelerating shock reversal [86]. The RCT on 7-day treatment with hydrocortisone and 9-alpha-fludrocortisone in patients with septic shock showed better outcomes in septic shock-associated early ARDS non-responders to short corticotrophin test, but not in responders and not in septic shock patients without ARDS [111] (Table 1). 

In agreement with these findings are also the data from meta-analyses performed on CS use in sepsis. In 2009, the systematic review of data from RCTs of CS vs. placebo or supportive treatment in adult patients with severe sepsis/septic shock demonstrated a decrease in 28-day mortality, increased 28-day shock reversal and reduced ICU duration of stay without increasing the risk of gastroduodenal bleeding, superinfection, or neuromuscular weakness. However, CS increased the risk of hyperglycemia and hypernatremia [112]. In 2015 after update of the previous reviews, authors identified 33 eligible trials in total (*n* = 4268 participants) evaluating the effects of CS in sepsis which confirmed that a long course of low-dose CS reduced 28-day mortality without inducing major complications but led to an increase in metabolic disorders [113].

Recently, several RCTs on the use of CS in sepsis have been published. The RCT by Tongyoo et al. has demonstrated hydrocortisone (50 mg every 6 h) for beneficial also in adult patients with early sepsis-associated ARDS (*n* = 98), where it improved PaO_2_ and LIS, and did not increase a rate of adverse events except of hyperglycaemia. However, it had no influence on day 28 survival [89] (Table 1).

Contrary, double-blind RCT including adult patients with severe sepsis (*n* = 190 CS-treated and *n* = 190 placebo) showed that hydrocortisone at a dose of 200 mg given as a continuous infusion for five days followed by dose tapering until day 11 did not reduce the risk of septic shock within 14 days nor reduced mortality in ICU or in the hospital, or mortality at 28 days [90] (Table 1).

Additional information has brought a multi-centre, double-blind RCT, in which hydrocortisone-plus-fludrocortisone therapy decreased 90-day mortality and mortality rate at ICU discharge and hospital discharge. Hydrocortisone-plus-fludrocortisone group had higher number of vasopressor-free days to day 28 and the number of organ-failure-free days. The rate of serious adverse events did not differ, but hyperglycaemia was more common in hydrocortisone-plus-fludrocortisone group [91] (Table 1).

Venkatesh et al. in RCT of patients with septic shock who received hydrocortisone (dose of 200 mg per day, *n* = 1832) for 7 days demonstrated faster resolution of shock, lower rate of blood transfusion, and shorter duration of the initial episode of mechanical ventilation, but without differences in ventilator-free days. There were no differences with respect to the mortality at 28 and 90 days, rate of recurrence of shock, number of days alive and out of the ICU or the hospital, recurrence of mechanical ventilation, rate of renal-replacement therapy, and the incidence of new-onset bacteraemia or fungemia [92] (Table 1).

Data from RCTs on CS use in septic shock has been recently summarized in two meta-analyses which, however, have led to distinct results. A systemic review of 22 RCTs including 7297 participants comparing low-dose CS to placebo in adults with septic shock from 2018 showed no effect on short- and longer-term mortality, increased rate of adverse events, but reduced duration of shock, mechanical ventilation and ICU stay [114]. Contrary, in more recent meta-analysis of outcome of adult patients with sepsis including 37 RCTs (*n* = 9564 patients), the CS treatment reduced 28-day mortality, ICU mortality and in-hospital mortality, increased shock reversal by day 7 and vasopressor-free days, but increased the risk of hyperglycaemia and hypernatremia [115].

### 3.4. Limitations

A majority of the above-mentioned animal studies have resulted into clear improvement of respiratory parameters and mitigation of lung injury. Data from several clinical trials has also shown a positive response to CS indicated by enhanced oxygenation, decreased mortality, increased number of ventilator-free days, however, some studies have exerted no significant benefit from the use of CS. In addition, delivery of CS can be associated with a variety of adverse effects which can decrease a value of this treatment. These issues potentially limiting the wider use of CS are discussed in the following subsections.

#### 3.4.1. Animal vs. Human Studies

Existing gaps in understanding the pathophysiology of ARDS and unsatisfactory response to treatment force the researchers to look for unknown interactions between the pathomechanisms and for testing of novel therapies. In this effort, data from in vivo animal models can be exceptionally valuable since some mechanisms of the injury can be hardly tested in humans but can be rather easily tested in the laboratory animals [116]. Animal models can reliably reproduce acute damage to the epithelial and endothelial barriers and acute lung inflammation, but no animal model reproduces all the characteristics of ARDS in humans, and most of animal models are relevant only for limited aspects of human ARDS. The highest value for transfer to clinical practice have the models where the injury is evolving over days or weeks, however, the prolonged support of ALI-injured animals is extremely technically difficult. In addition, human lungs can be hit by the mechanisms of the primary illness (e.g., pneumonia or sepsis), but the course and prognosis are influenced by hereditary factors, susceptibility to the triggering agents, concomitant diseases, age etc. as well as by therapies used for supportive care (e.g., mechanical ventilation) [116]. In contrast to heterogeneous and highly variable combination of pathogenetic factors in individual patients, interaction of the mentioned factors is limited in animals which enter the study as healthy, well-fed, usually young animals in good shape and of the comparable age and body weight. Because of strictly homogenous distribution of animals into the study groups and keeping the same study design, results of the animal studies appear to show lower inter-individual differences. Therefore, the effects of administered therapy can be found as statistically significant in animal studies while delivery of the same therapeutic agent in humans can lead to other results. However, different response to the treatment can also be caused by the inter-species differences, e.g., species differences in an innate immune response (differences in Toll-like receptors, in a mononuclear phagocyte system, in a production of NO, in chemokines and chemokine receptors) as well as differences in an animal size considered in disease modelling [116]. The above-mentioned animal vs. human differences reduce a direct clinical applicability and a prediction value of animal experiments for clinical studies. Nevertheless, studies on animal models represent an essential and indispensable tool for better understanding the pathophysiology of ARDS and for development and testing of promising therapeutic strategies in the pre-clinical conditions.

#### 3.4.2. Human vs. Human Studies

The existence of rather large differences between the individual clinical studies is complex, resulting from contribution of several factors. Among the published studies, there are differences in the aetiology of ARDS (pulmonary or extrapulmonary). As mentioned in the Introduction, in direct or pulmonary ARDS the noxious agent primarily hits the epithelial lung cells, while the endothelial damage and systemic inflammation are typical for extrapulmonary ARDS. Importantly, differences in primarily hit tissue and related mechanisms of injury may be also responsible for variable response for treatments [21,22,23]. Furthermore, effectiveness of the given therapy strongly depends on the stage of the disease. Results from recent RCTs by Meduri’s group indicate that administration of CS in early ARDS leads to better response than their administration in persistent ARDS (later than 14 days from the impact). Other important factor responsible for different results are the differences in entry criteria of the individual studies, age of patients, respiratory mechanics, degree of inflammatory response, used ventilatory strategies etc. Finally, various dosing and choice of CS preparates with different glucocorticoid and mineralocorticoid potencies and plasma and biological halftimes can lead not just to different therapeutic response, but also to different adverse effects [117]. Large heterogeneity in the mentioned parameters then significantly alters the final outcome and limits the comparability among the studies.

#### 3.4.3. Adverse Effects of CS

In addition to potent anti-inflammatory, anti-oedematous and pulmonary vasodilation effects which may be of benefit in ARDS, CS also exert a variety of side effects. As stress hormones, release of CS via activation of the hypothalamic-pituitary-adrenal axis recruits glucose to supply energy to organs facing stress, leading to arousal reactions and immune responses to maintain homeostasis. CS have opposite effects to insulin increasing turnover between the stored energy (in glycogen, triglycerides and protein) and freely available fuel for mitochondrial oxidation (glucose and free fatty acids) [118]. Therefore, long-term stimulation or administration of excessive CS may lead to protein catabolism, gluconeogenesis and glucogenesis resulting in hyperglycaemia, and other changes such as hypokalaemia, dyslipidaemia, reduced fibrinolysis, posterior subcapsular cataract, exacerbation of glaucoma, increased intracranial pressure, peptic ulcers, upper gastrointestinal bleeding, immunosuppression, neuropsychiatric disturbances, osteoporosis, myopathy, irregularities of the menstruation cycle, etc. [119]. CS also affect the cardiovascular parameters whereas the raise in blood pressure is partially mediated by renal sodium retention and plasma volume expansion. In addition, both glucocorticoid and mineralocorticoid receptors are expressed in the heart and arterial walls, where CS act directly to maintain vascular tone and modify vascular inflammatory, proliferative and remodelling responses to injury [118]. Nevertheless, adverse effects of CS appear more likely after the long-term treatment, but less-frequently after the short-term treatment, even with high CS doses. It might be explained by time-dependent genomic effects of CS, which do not elevate further after high doses, when GR saturation is already achieved [120].

In ARDS, acute adverse effects may occur within several days of initiation of the CS therapy. In critically ill patients, muscle weakness or acute myopathy have been reported after the treatment with CS or CS in combination with NMBA [121,122,123]. Development of acute CS myopathy is rather heterogeneous. Myopathy can arise within 1–3 days after delivery of a single dose or repetitive doses of CS, but there is no clear association between the dose and/or route of administration and the occurrence of myopathy. The muscle weakness most often occurs on proximal limbs, but distal limbs, bulbar and respiratory muscles may also be affected. Cessation of CS usually leads to improvement, but rarely the status can be irreversible [123]. Therefore, when given concurrently with CS, NMBA should be avoided to minimize the risk of neuromuscular weakness [50,121,122,123]. Administration of CS may be associated with allergic or anaphylactic reactions. Particularly the patients who received repetitive doses of CS can develop various forms of hypersensitivity, most commonly anaphylaxis, urticaria, and angio-oedema [117,124,125]. CS also blunt the febrile response; therefore, infection surveillance should be carefully made [50]. Furthermore, CS therapy can be linked with a variety of neuropsychiatric complications, such as acute mania or depression, psychosis, or delirium [126,127]. Other known side effects of administration of CS are hypokalaemia and increased risk of peptic ulcer disease [117,128]. After CS therapy, hyperglycaemia is also frequently seen [117,128], as it was referred in several RCTs [88,89,90,91], as well. To minimize glycaemic variations, CS could be administered as a continuous infusion [50,129,130]. 

After a complete course, slow CS dosage reduction within 9–12 days should be made to allow recovery of GR number and HPA axis. Rapid discontinuation of CS may increase the risk of rebound inflammation, readmission to the ICU, reinstitution of mechanical ventilation, or even increased mortality [50,51]. Slow weaning and tapering the dose of CS in ARDS or sepsis have been used in several studies [83,85,87,90]. For methylprednisolone treatment, Meduri et al. published weaning protocols in both early severe ARDS and late unresolving ARDS [50].

Special attention should be paid in the paediatric patients with ARDS, with respect particularly to rebound effects after CS discontinuation, risk of nosocomial infections, influence on bone growth and child development, consequences on vaccination and long-term systemic morbidity including muscle weakness, impaired physical function and neurocognitive dysfunction [51].

## 4. Concluding Remarks

CS possess a very large therapeutic potential in ARDS because of their potent anti-inflammatory, anti-oedematous, pulmonary vasodilator and other actions. In spite of expectations, results of both clinical and experimental studies on the use of CS in ALI/ARDS or sepsis are often contradictory. These discrepancies may originate from large heterogeneity in terms of patients inclusion criteria, severity of ARDS at the moment of CS delivery, timing, dosing and duration of CS, choice of CS agent etc. [131]. 

Considering the differences between the pathophysiological changes and responses to CS, different recommendations have been made for early and late ARDS. For instance, in patients with early severe ARDS, methylprednisolone at a dose of 1 mg/kg per day given as an infusion and tapered over four weeks can be associated with a favourable risk-benefit profile. For patients with unresolving ARDS, methylprednisolone at a dose of 2 mg/kg per day initiated before day 14 of ARDS and continued for at least two weeks following extubation may bring some positive effects. However, if treatment starts after day 14, the CS treatment can exert no significant benefit [50,83,85,87]. For sepsis, hydrocortisone was the most commonly used CS agent, at a dose of 200–300 mg/day given as an infusion (with a bolus of 50–100 mg given before infusion) or as boluses every 6 h, typically for 7–14 days [110,130]. Prolonged low-dose CS therapy initiated within the first two weeks may also be beneficial in selected patients with pediatric ARDS [51,132,133]. Then, the CS dose should be slowly tapered to avoid or minimize the above-mentioned complications [50]. In general, CS are relatively safe drugs with low risk profile when secondary prevention measures (appropriate glycaemic control, minimized use of sedation and NMBA, control of infection and monitoring of ventilatory and cardiovascular parameters) are implemented [50]. Then the benefits of this low-cost therapy, which is worldwide familiar for doctors, may overcome the eventual risks [50]. However, before any recommendations can be made, RCT trials on the prolonged use of CS in specific subgroups of adult and paediatric ARDS patients should be carried on, defining safety profile and potential side effects [51].

In conclusion, data from experimental and clinical studies indicate that CS have a potential to improve the lung function, alleviate inflammation, and enhance survival in acute lung damage. However, there is an urgent need for additional RCTs in adult and paediatric patients with ARDS to bring more information on the optimized CS therapeutic protocol.

## Figures and Tables

**Table 1:** Randomized controlled trials of CS in adult patients with ARDS or sepsis.

Author (Year)	Diagnosis	Total No. of Patients (CS/Placebo)	Treatment/Dose	Duration of Therapy (Days)	Outcomes in CS Groups
Bernard et al. (1987) [82]	Early ARDS	99 (50 CS/49 placebo)	Methylprednisolone (30 mg/kg 6-hourly)	1	No differences in mortality, infectious complications or ventilatory characteristics 5 days after entry
Meduri et al. (1998) [83]	Severe persistent ARDS	24 (16 CS/8 placebo)	Methylprednisolone (2 mg/kg loading dose, then 2 mg/kg/day for days 1–14, 1 mg/kg/day for days 15–21, 0.5 mg/kg/day for days 22–28, 0.25 mg/kg/day for days 29–30, and 0.125 mg/kg/day for days 31–32)	14	Improvements in LIS and PaO_2_/FiO_2_ Reduced ICU and hospital mortality No increase in infection complications
Confalonieri et al. (2005) [84]	Severe community-acquired pneumonia	46 (23 CS/23 placebo)	Hydrocortisone (bolus 200 mg, then infusion 10 mg/hour)	7	Improvement in PaO_2_/FiO_2_ and chest X-ray score Reduction in CRP levels, MODS score, and delayed septic shock Reduction in length of hospital stay and mortality
Steinberg et al. (2006) [85]	Persistent ARDS	180 (89 CS/91 placebo)	Methylprednisolone (2 mg/kg loading dose, then 0.5 mg/kg 6-hourly for 14 days, 0.5 mg/kg 12-hourly for 7 days)	14	Starting CS therapy later than 2 weeks after the onset of ARDS associated with increased mortality
Annane et al. (2006) [86]	Septic patients with ARDS	177 (85 CS, including 23 responders/92 placebo, including 25 responders)	Hydrocortisone (50 mg 6-hourly) and 9-α- fludrocortisone (50 mg once a day)	7	In nonresponders to short corticotrophin test: decreased mortality and more ventilator days off, no difference in responders
Meduri et al. (2007) [87]	Early severe ARDS	91 (63 CS/28 placebo)	Methylprednisolone (1 mg/kg loading dose, then 1 mg/kg/day for days 1–14, 0.5 mg/kg/day for days 15–21, 0.25 mg/kg/day for days 22–25, 0.125 mg/kg/day for days 26–28)		Shorter duration of mechanical ventilation Reduced ICU stay and ICU mortality No increase in infectious complications
Meijvis et al. (2011) [88]	Community-acquired pneumonia	304 (151 CS/153 placebo)	Dexamethasone (5 mg once a day)	4	Shorter length of stay No differences in hospital mortality or severe adverse events More common hyperglycaemia
Tongyoo et al. (2016) [89]	Severe sepsis or septic shock	197 (98 CS, 99 placebo)	Hydrocortisone (50 mg 6-hourly)	7	Improvement in PaO_2_/FiO_2_ and LIS No survival benefit More frequent hyperglycaemia
Keh et al. (2016) [90]	Severe sepsis	380 190 CS/190 placebo)	Hydrocortisone (200 mg continuous infusion for 5 days, then dose tapering until day 11)	5	No reduction of risk of septic shockNo differences in mortality in ICU or in the hospital Higher occurrence of secondary infections, muscle weakness, and hyperglycemia
Annane et al. (2018) [91]	Septic shock	1241 (614 CS/627 placebo)	Hydrocortisone (50 mg 6-hourly) and 9-α- fludrocortisone (50 mg once a day)	7	Lower 90-day mortality More vasopressor-free days and organ-failure-free days to day 28 No difference in ventilator-free days and rate of serious adverse events More common hyperglycemia
Venkatesh et al. (2018) [92]	Septic shock	3658 (1832 CS/1826 placebo)	Hydrocortisone (200 mg per day)	7	No improvement in 90-day mortality Faster resolution of shock Fewer blood transfusions Shorter duration of initial mechanical ventilation No difference in ventilation-free days

**Table 2:** Randomized controlled trials of CS in paediatric patients with ARDS.

Author (Year)	Diagnosis	Total No. of Patients (CS/Placebo)	Treatment/Dose	Duration of Treatment (Days)	Outcomes in CS Groups
Drago et al. (2015) [107]	Paediatric ARDS	35 (17 CS, 18 placebo)	Methylprednisolone (loading dose 2 mg/kg, then 1 mg/kg/day infusion)	7	No differences in length of mechanical ventilation, ICU stay, hospital stay, or mortality Lower PaCO_2_ on days 2 and 3, higher pH on day 2, and higher PaO_2_/FiO_2_ on days 8 and 9 Lower requirement for treatment of postextubation stridor or supplemental oxygen at ICU transfer No adverse effects
Schwingshackl et al. (2016) [108]	Paediatric ARDS	35 (17 CS, 18 placebo)	Methylprednisolone (loading dose 2 mg/kg, then 1 mg/kg/day infusion)	7	On day 7, increased WBC and platelets counts, lower IFN-α, IL-6, IL-10, MCP-1, G-CSF and GM-CSF levels, and higher IL-17α levels in comparison to study entry
Kimura et al. (2016) [109]	Paediatric ARDS	35 (17 CS, 18 placebo)	Methylprednisolone (loading dose 2 mg/kg, then 1 mg/kg/day infusion)	7	On day 7, reduction in MMP-8 levels, no increases in sICAM-1, on day 8 positive correlation of sRAGE levels with PaO_2_/FiO_2_, negative correlation of O_2_ requirements at ICU transfer with day 7 sICAM-1 levels

## Abbreviations

ACTH	Adrenocorticotropic hormone
ALI	Acute lung injury
AP-1	Activator protein-1
ARDS	Acute respiratory distress syndrome
ATP	Adenosine triphosphate
BALF	Bronchoalveolar lavage fluid
CAP	Community-acquired pneumonia
cGCR	Cytosolic glucocorticoid receptor
CRP	C-reactive protein
CS	Corticosteroids
DAMPs	Danger-associated molecular patterns
DNA	Deoxyribonucleic acid
eNOS	Endothelial nitric oxide synthase
FiO2	Fraction of inspired oxygen
G-CSF	Granulocyte colony-stimulating factor
GM-CSF	Granulocyte macrophage colony-stimulating factor
GR	Glucocorticoid receptor
GRE	Glucocorticoid response element
HCAP	Healthcare-associated pneumonia
i.p.	Intraperitoneal
i.t.	Intratracheal
i.v.	Intravenous
ICAM	Intercellular adhesion molecule
ICU	Intensive care unit
IFN	Interferon
IL	Interleukin
LIS	Lung injury score
LPS	Lipopolysaccharide
MCP	Monocyte chemoattractant protein
MIP	Macrophage inflammatory protein
MMP	Matrix metalloproteinase
MODS	Multiple organ dysfunction syndrome
MPO	Myeloperoxidase
mRNA	Mitochondrial ribonucleic acid
NETs	Neutrophil extracellular traps
NF-κB	Nuclear factor-κB
NMBA	Neuromuscular blocking agents
NO	Nitric oxide
NOS	Nitric oxide synthase
PaCO2	Arterial partial pressure of carbon dioxide
PaO2	Arterial partial pressure of oxygen
PEEP	Positive end-expiratory pressure
RAGE	Receptor for advanced glycation end products
RCT	Randomized controlled trial
SIRS	Systemic inflammatory response syndrome
TGFβ	Transforming growth factor
TNF	Tumour necrosis factor
VILI	Ventilator-induced lung injury
V_T_	Tidal volume
vWf	Von Willebrand factor
WD	Wet-dry lung weight ratio
